# Real‐World Usage of CaHA‐CMC:CPM‐HA Hybrid Dermal Fillers: Findings From a Delphi Panel

**DOI:** 10.1111/jocd.70688

**Published:** 2026-01-30

**Authors:** Jonathan Kadouch, Rolf Bartsch, Nabil Fakih‐Gomez, Omar Haroon, Martina Kerscher, Luiz Perez, Rossana Vasconcelos, Yana Yutskovskaya, Carla de Sanctis Pecora

**Affiliations:** ^1^ ReSculpt Clinic Amsterdam the Netherlands; ^2^ The Aesthetics Vienna Austria; ^3^ Department of Facial Plastic and Cranio‐Maxillo‐Facial Surgery Fakih Hospital Khaizaran Lebanon; ^4^ Facexpert Clinic and Academy Torremolinos Spain; ^5^ Praxis Dr Omar Haroon Zürich Switzerland; ^6^ Division of Cosmetic Sciences University of Hamburg Hamburg Germany; ^7^ Espaço Mira Private Clinic São Paulo Brazil; ^8^ Nomina Clinic University of Santo Amaro São Paulo Brazil; ^9^ Department of Dermatology and Venereology and Cosmetology Pacific State Medical University Moscow Russia; ^10^ Dermatologie Clinic São Paulo Brazil

**Keywords:** aesthetics, calcium hydroxylapatite, cohesive polydensified matrix‐hyaluronic acid, Delphi methodology, dermal fillers, guidelines, hybrid dermal fillers

## Abstract

**Background:**

The popularity of minimally invasive aesthetic procedures has increased globally, with a 40.9% increase in non‐surgical procedures between 2019 and 2023. Dermal fillers have gained popularity for delivering visible results with short recovery times. Among these, hybrid dermal fillers combining calcium hydroxylapatite‐carboxymethyl cellulose (CaHA‐CMC) and cohesive polydensified matrix‐hyaluronic acid (CPM‐HA) offer tailored treatment with demonstrated efficacy and tolerability. However, standardized guidance for use remains limited.

**Objective:**

To establish consensus among a panel of aesthetic healthcare professionals (HCPs) on the real‐world use of CaHA‐CMC:CPM‐HA hybrid dermal fillers.

**Methods:**

A modified Delphi process was conducted with 23 international HCPs. Statements were developed from scientific advisory board outcomes and a literature review, refined by a scientific steering committee and evaluated across three rounds. Consensus was defined as ≥ 80% agreement/disagreement.

**Results:**

Consensus was reached on the safety, efficacy, and use of CaHA‐CMC:CPM‐HA hybrid dermal fillers for volumization, contouring, and skin quality improvement. Of 43 statements, 27 reached consensus in Round 1, 7 in Round 2, and 3 in Round 3. Recommendations included dilution ratios, the “foaming” technique, subdermal injection, and avoiding high muscle activity sites. Experts highlighted the synergistic effects and convenience of CaHA‐CMC:CPM‐HA fillers, while noting the need for further research on long‐term safety and rheological properties.

**Conclusion:**

These results provide a base for standardized hybrid dermal filler use. Agreement on topics including indications, efficacy, and safety underscore the experts' unified stance on hybrid dermal filler usage. These consensus statements can serve as guidelines to ensure effective and safe clinical practice.

## 1 Introduction

The population and adoption of minimally invasive aesthetic procedures as alternatives to plastic surgery have increased globally; specifically, there has been a 40.9% increase in non‐surgical procedures from 2019 to 2023 [1, 2]. Among these, dermal fillers have gained popularity due to their ability to provide noticeable improvements with short recovery times, at a low cost, supported by long‐term safety data [3]. Fillers are typically indicated for facial rejuvenation and skin quality improvements, volumization, and facial contour augmentation [4, 5]. Fillers differ in their modes of action and tissue interactions. For example, calcium hydroxyapatite (CaHA) fillers provide immediate volume through their gel carrier and stimulate long‐term collagen and elastin production via direct mechanical interactions. Hyaluronic acid (HA) fillers, on the other hand, offer a wide range of applications, ranging from immediate volumization to skin hydration, depending on their physical properties and manufacturing process [4, 5]. Hybrid dermal fillers that combine CaHA with HA in one syringe have become increasingly popular in aesthetic medicine, demonstrating both efficacy and a good tolerability profile [6, 7]. In most published literature focused on hybrid dermal fillers, the HA component has been one of the cohesive polydensified matrix HA fillers (cohesive polydensified matrix‐hyaluronic acid; CPM‐HA [Belotero collection, Anteis S.A., Plan‐les‐Ouates, Switzerland, a company of the Merz Aesthetics group]). The use of calcium hydroxylapatite‐carboxymethylcellulose (CaHA‐CMC [Radiesse, Merz North America Inc., Franksville, WI]) and CPM‐HA as a hybrid combination (CaHA‐CMC:CPM‐HA) for the improvement and restoration of facial shape and contouring has been demonstrated to provide effective volumizing and lifting following injections of the cheeks and jawline [8, 9]. Compared with commercially available premixed hybrid dermal fillers, the option of mixing CaHA‐CMC with formulations of CPM‐HA, each of which have different concentrations of HA and distinct rheologies, allows for a flexible and tailored treatment according to the desired outcome, anatomical area, and individual patient needs [10].

Although the evidence supporting of dermal fillers is established, guidelines on the use of these products in combination as hybrid dermal fillers remains limited. Further research is therefore needed to evaluate their efficacy, safety, and durability, and to establish standardized protocols for their clinical application. As with any relatively novel treatment, there is a need to establish expert consensus to inform best practices within the clinic, and to determine and address any gaps within the published literature; this applies equally to the application of CaHA‐CMC:CPM‐HA hybrid dermal fillers. To this end, nine international experts from seven countries convened in an advisory board meeting which aimed to gain practical insights on the use of hybrid dermal fillers in clinical practice. This meeting underscored the need for consensus on a range of topics, including indications, efficacy, administration techniques, injection region, perceived patient benefit, safety, and contraindications. The Delphi panel methodology was proposed in this meeting as the optimal approach for achieving this consensus (*Merz advisory board, 2024, unpublished data*).

The primary objective of this Delphi panel study was, therefore, to establish consensus among a panel of aesthetic healthcare professionals (HCPs) regarding the real‐world use of CaHA‐CMC:CPM‐HA hybrid dermal fillers. In establishing this consensus, the aim was to provide expert‐led and standardized guidance on the use of hybrid dermal fillers. Secondary objectives included reaching agreement on the basic safety and efficacy requirements for CaHA‐CMC:CPM‐HA hybrid dermal fillers in volumization, contouring, and skin quality improvement, as well as identifying current gaps in the scientific evidence to guide future use of these hybrid dermal fillers.

## 2 Methods

### 2.1 Study Design

This study utilized a modified Delphi panel design, across three rounds, consisting of two online surveys and one virtual working session, all conducted entirely in English. Delphi panel methodology is a systematic and structured process to achieve consensus among a panel of experts within a given field, which is used widely in health sciences research [11].

### 2.2 Study Participants

The Delphi panel consisted of aesthetic HCPs with experience in the use of hybrid dermal fillers in different aesthetic indications. Panelists were recruited from a list provided by the study sponsor to ensure that all had previous experience of using the CaHA‐CMC:CPM‐HA hybrid dermal fillers being discussed. Panelists needed to meet all the following criteria to be eligible for inclusion in the study: specialty in aesthetic medicine and experience in treating aesthetic conditions; good knowledge of CaHA‐CMC:CPM‐HA hybrid dermal filler; regular use of CaHA‐CMC:CPM‐HA hybrid dermal filler in their daily practice; and proficiency in English. Panelists were recruited from Europe, the Middle East, and Latin America, as these countries had product availability and the greatest experience with CaHA‐CMC:CPM‐HA hybrid dermal fillers at the time of the study. Selection of panelists from these countries ensured that all participants were able to contribute to the panel discussions based on substantial hands‐on expertise.

### 2.3 Study Procedures

An advisory board organized by the study sponsor in 2024 aimed to establish clear indications and guidance with respect to the safe, optimal use of CaHA‐CMC:CPM‐HA hybrid dermal fillers in clinical practice and to identify evidence‐generation gaps required to support this guidance. During this meeting, the faculty provided expert insights and identified key topics for inclusion in the Delphi panel study to assess additional treatment considerations discussed during the meeting. Based on insights gathered during the advisory board meeting, along with findings from an initial literature review, a set of evidence‐based statements was developed to describe real‐world practice in the use of CaHA‐CMC:CPM‐HA hybrid dermal fillers. The literature review focused on understanding the practical clinical recommendations for the use of CaHA‐CMC:CPM‐HA hybrid dermal fillers and identifying potential evidence‐generation gaps required to support the guidance.

A scientific steering committee comprised of three experts from the Netherlands, Austria, and Brazil, each with extensive experience in the use of hybrid dermal fillers for aesthetic indications, was onboarded to review and approve the study protocol, research materials, and statements for the Delphi panel consensus, and to contribute to evidence generation by reviewing the analyses, results, and statement recommendations. The scientific steering committee also supported the refining of statements between Delphi panel rounds based on input provided by the Delphi panel members. Prior to the initiation of Round 1 of the Delphi panel, statements were further refined via 60‐min in‐depth pilot interviews conducted with two aesthetic dermatologists experienced in the use of hybrid dermal fillers for aesthetic indications. This provided independent review and validation of the draft statements to ensure appropriate wording for clarity, inclusion of essential discussion points, and intuitive ordering to aid conversation.

To mitigate potential bias, the study was designed and conducted in strict adherence to internationally recognized market research codes and ethical standards, including those set forth by the European Pharmaceutical Market Research Association, the British Healthcare Business Intelligence Association, the Market Research Society, and the European Society for Opinion and Marketing Research, all of which emphasize transparency, respondent confidentiality, and the avoidance of leading or suggestive questioning.

**FIGURE 1:**
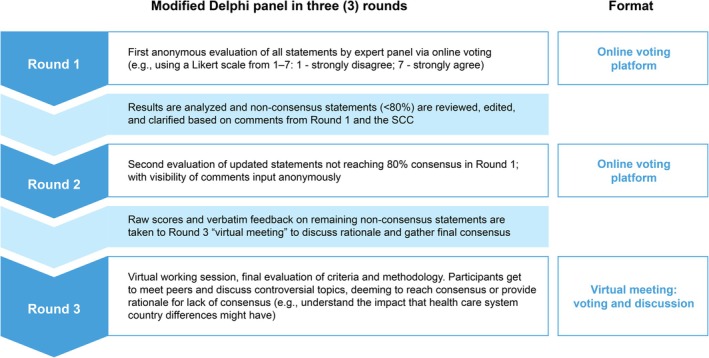
Modified Delphi panel rounds design. SCC, scientific steering committee.

A three‐round modified Delphi panel design was used (Figure 1). For Round 1, each panelist was invited to visit an online platform over a period of 7 days and express their level of agreement with the full list of statements presented as per their identified categories. Each panelist could suggest improvements in free text fields for statements where they neither agreed nor disagreed. Statements reaching consensus were removed, and those which did not reach consensus were refined in collaboration with the scientific steering committee. In Round 2 only the refined statements based on those that had not reached consensus were presented for voting, using the same process as in Round 1 with the online platform available for a period of 10 days. Statements not reaching consensus were further refined for consideration in Round 3. Round 3 consisted of a 2‐h online workshop session where Delphi panel members met to discuss and refine those statements that had previously failed to reach consensus.

### 2.4 Definitions of Consensus

The Delphi panel process ensured that the final recommendations were reflective of collective expert agreement, providing a robust foundation for decision‐making. The level of agreement/disagreement required for consensus Delphi panel studies is typically set at 70%–80% [12]. To ensure the robustness of the findings of this study, consensus was considered to be reached when ≥ 80% of the panel agreed or disagreed with a statement.

The study was conducted in accordance with legal and regulatory requirements, as well as with scientific purpose, value, and rigor, following generally accepted research practices. This study was submitted to an Institutional Review Board/Independent Ethics Committee to obtain an exemption determination, given that no patient data would be collected, no medicinal product was being researched, and data would be collected through an online survey and interviews.

## 3 Results

### 3.1 Study Participants

Overall, 23 HCPs were invited to participate as members of the Delphi panel, with 22 of these (95.7%) completing Round 1. Subsequently, these 22 participants were invited to participate in Round 2 of the study with 21 HCPs completing Round 2. Six HCPs participated in the online workshop conducted for Round 3 of the Delphi panel (Table 1). The limited number of HCPs available for Round 3 was driven in part by the format of this round, an online workshop at a defined time rather than an online survey available over several days.

**TABLE 1:** Number of participants per geographic location across Delphi panel rounds.

Region	Round 1	Round 2	Round 3
Europe and Middle East	17	16	3
Latin America	5	5	3
Total	22	21	6

### 3.2 Statement Categories

Expert opinions gathered during the 2024 Advisory Board Meeting informed the categories of statements examined by the Delphi panel. The advisory board faculty advocated the need to reach consensus regarding the administration of hybrid dermal fillers. Given the study objectives, statements categories focused on nomenclature (one statement); indications and efficacy (10 statements); administration techniques (13 statements); injection region (six statements); perceived patient benefit (three statements); safety and contraindications (six statements); and evidence gaps (four statements).

### 3.3 Statement Consensus

**FIGURE 2:**
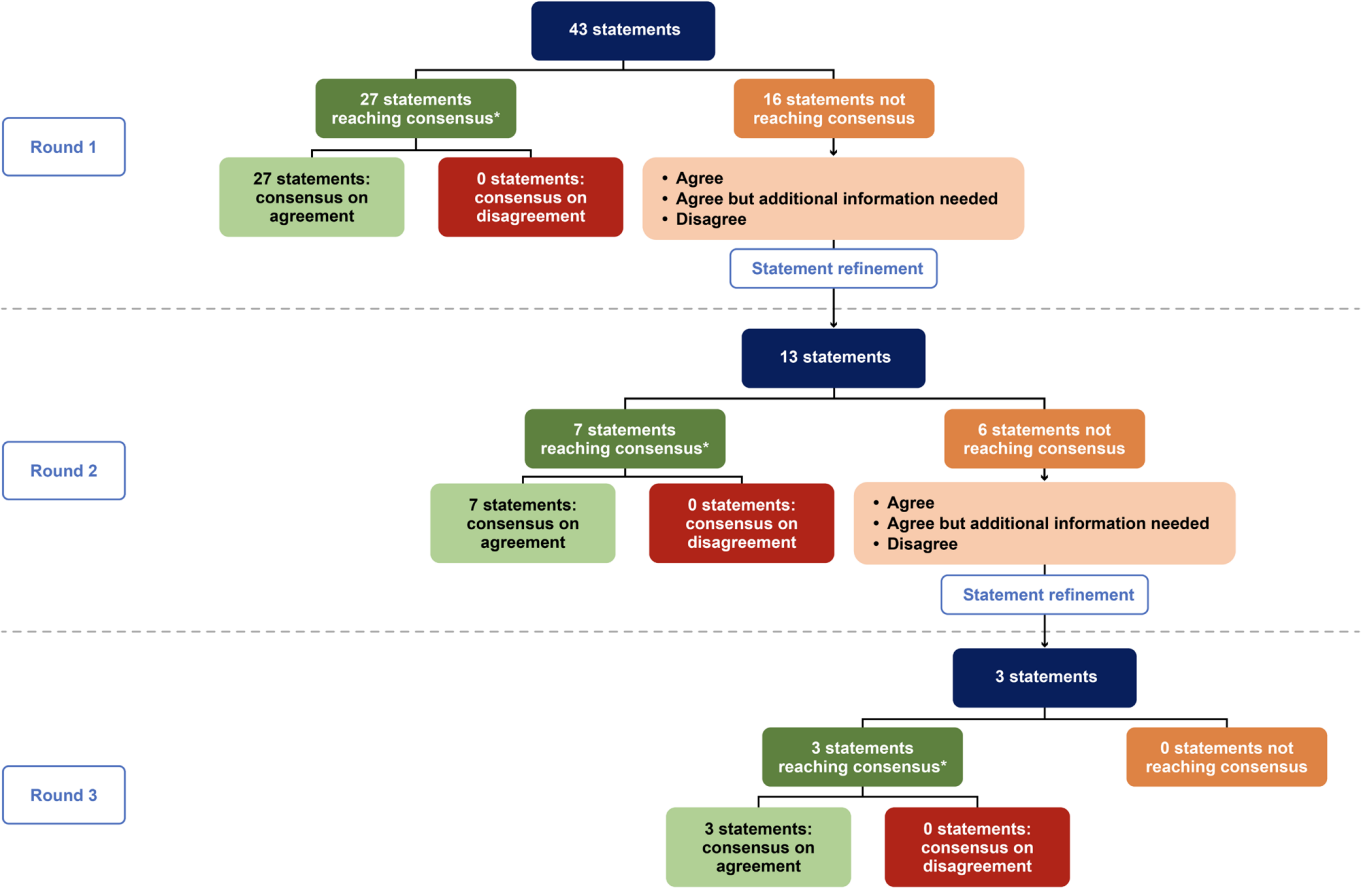
Summary of consensus at Round 1, Round 2, and Round 3 of the Delphi panel. *≥ 80% responses agreeing or disagreeing with a statement.

Overall, 43 statements were examined in Round 1, with 27 statements (62.8%) reaching consensus (Figure 2). In Round 2, 13 statements, which had been refined from the 16 statements that did not reach consensus in Round 1 were examined, seven of which reached consensus (53.8%; Figure 2). Following statement refinement, three statements from the six that did not reach consensus in Round 2 were taken to Round 3. Of these three statements, one reached consensus during the first round of voting. The panel discussed the remaining two statements with the HCPs encouraged to share their opinions and suggest rephrasing. After rephrasing to capture all expressed opinions, these two statements also reached consensus (Figure 2). Details of the statements within each category, together with the stage at which they reached consensus, are provided in Table 2.

**TABLE 2:** Consensus statements for the use of hybrid dermal fillers.

Category	Statements	Round 1	Round 2	Round 3
Nomenclature	“Hybrid Concentrate” (no additional “fluid”) for 1 syringe CaHA‐CMC (1.5 mL) + 1 syringe CPM‐HA (1.0 mL)			✓
	“Hybrid” (0.5 mL “fluid”) for 1 syringe CaHA‐CMC (1.5 mL) + 1 syringe CPM‐HA (1.0 mL) + lidocaine (0.5 mL)			✓
	“Hybrid Dilute” (1.5 mL “fluid”) for 1 syringe CaHA‐CMC (1.5 mL) + 1 syringe CPM‐HA (1.0 mL) + lidocaine (0.5 mL) + saline (up to 1.0 mL)			✓
	“Hybrid Hyperdilute” (more than 1.5 mL “fluid”) for 1 syringe CaHA‐CMC (1.5 mL) + syringe CPM‐HA (1.0 mL) + lidocaine (0.5 mL) + saline (at least 2.5 mL)			✓
Indications and efficacy	Hybrid dermal fillers CaHA‐CMC:CPM‐HA are used to provide volume to tissues, rejuvenate, reshape localized areas (contouring), tightening neck and hand, and improve skin quality	✓		
	The combination of CaHA‐CMC and CPM‐HA dermal filler has a synergistic effect, both immediately and in the long term	✓		
	When using CaHA‐CMC and CPM‐HA in combination, CPM‐HA filler compensates for the early volume loss of CaHA filler due to the reabsorption of the CMC	✓		
	CaHA‐CMC add neocollagenesis properties to a CPM‐HA filler, whereas a high G′ CPM‐HA can enhance a CaHA‐CMC filler by adding additional volumization while securing tissue softness	✓		
	The dilution of CaHA‐CMC:CPM‐HA formulation in saline or lidocaine decreases the G′ and viscosity of the original formulation, making it softer and easier to inject	✓		
	Addition of lidocaine to CaHA‐CMC:CPM‐HA is used by clinicians to provide more comfort for the patient, while also increasing smoothness and malleability			✓
Administration techniques	For volumization, a 1:1 CaHA‐CMC:CPM‐HA 26 mg/mL hybrid mixture (with 0.5 mL of lidocaine added to the mix) may be administered		✓	
	For contouring, a 1:1 CaHA‐CMC:CPM‐HA 25.5 mg/mL hybrid mixture (with 0.5 mL of lidocaine added to the mix) may be administered		✓	
	The use of CaHA‐CMC:CPM‐HAG 20 mg/mL hybrid hyperdilute (addition of 1.5–4.5 mL saline) is a suitable filler to improve skin quality	✓		
	To achieve a homogenous hybrid dermal filler CPM‐HA, CaHA‐CMC and lidocaine (ratio dependent on indication) need to be mixed 20 passes	✓		
	The “foaming” technique used to prepare the CaHA‐CMC:CPM‐HA hybrid dermal filler increases product homogeneity and helps with product distribution in the tissue	✓		
	For volumization hybrid dermal filler CaHA‐CPM‐HA 26 mg/mL is administered via a subdermal injection using a 22G or 25G cannula	✓		
	For contouring, hybrid dermal filler CaHA‐CMC:CPM‐HA 25.5 mg/mL is administered via subdermal injection using a 22G or 25G cannula	✓		

	For skin quality improvement, hybrid hyperdilute filler CaHA‐CMC:CPM‐HAG 20 mg/mL or CaHA‐CMC:CPM‐HA 22.5 mg/mL is administered via a subdermal injection in multiple directions using either a 22G or 25G cannula	✓		
	For cases with minimal or little volume loss and laxity, the CaHA‐CPM‐HA 26 mg/mL mix can be injected with 1:1 ratio for facial lifting to provide subtle enhancement while maintaining product efficiency	✓		
	When treating a more extensive volume loss (with limited laxity), the ratio of the CaHA‐CPM‐HA 26 mg/mL mixture increases up to 1:2 for facial volumization		✓	
	When treating a more extensive laxity, the ratio of the CaHA‐CMC:CPM‐HA mixture can be increased up to 2:1			✓
	For smaller contour corrections, CaHA‐CMC:CPM‐HA 25.5 mg/mL ratio of 1:1 is applied which is sufficient to achieve the desired aesthetic outcome without overcorrection	✓		
For skin quality improvements and areas of thin tissue envelopes (e.g., neck, décolleté), a CaHA‐CMC:CPM‐HAG 20 mg/mL or CaHA‐CMC:CPM‐HA 22.5 mg/mL ratio of 1:3 hyperdilute (i.e., 1.5 mL CaHA, 1.0 mL CPM‐HA, 0.5 mL lidocaine, which is further diluted with saline to reach 1:3 ratio, a total of 6.0 mL) may be applied	✓		
Injection region	Hybrid dermal fillers are best injected in the subcutaneous layer where the CaHA‐CMC microspheres can form a scaffold for fibroblast infiltration and encourage the formation of new collagen	✓		
	Injection areas with hybrid hyperdilute filler for skin quality improvement include the lower face, neck, decolletage, as well as the mid‐third, upper third, or the entire dorsum of the hand	✓		
	The facial treatment areas with hybrid dermal fillers include the volumization or contouring of temples, cheeks, preauricular region, and jawline		✓	
	Hybrid dermal fillers must not be used when injecting the nose, lips, or eyes	✓		
	The treatment areas with hybrid dermal fillers for sharp jawline contour include the posterior jaw angle, anterior jaw depression, chin, and jawline contour	✓		
	The treatment areas for neck lifting primarily include the anterior region of the neck, anterior to the sternocleidomastoid muscle (SCMM), including under chin, submental area, and jawline		✓	
Perceived patient benefit	CaHA‐CMC:CPM‐HA hybrid dermal fillers have an increased demand among clinicians to reduce facial wrinkles and improve facial volume and contours	✓		
	CaHA‐CMC:CPM‐HA hybrid dermal fillers are a convenient procedure because both fillers are administered in a single injection, and they decrease the need for retouching	✓		
	Hybrid dermal fillers CaHA‐CMC:CPM‐HA are used in men and women seeking a multidimensional effect to multiple tissue structures (or layers, as in skin and subdermal plane) to the aging face, that is, volume and/or rejuvenated skin	✓		
Safety and contraindications	It has been proven to be safe to mix CaHA‐CMC with CPM‐HA		✓	
	CaHA‐CMC and CPM‐HA are a safe combination for hybrid dermal filler	✓		
	Similar to CaHA‐CMC used alone, hybrid dermal fillers may not be advised for injection into areas with repetitive muscle activity, such as the perioral and periorbital regions	✓		
	In order to limit implant site nodules post‐hybrid filler injection, it is recommended to use a slow retrograde technique, avoid bolus injections, stop the injection prior to removal of the cannula (thus avoiding superficial deposition of product), and deliver all injections to the subdermal area	✓		
Evidence gaps	A lack of standardization in the application of hybrid dermal fillers leads to variations in treatment outcomes and an increased potential for complications	✓		
	While the safety of individual components is well‐established, the long‐term safety of hybrid dermal fillers (CaHA‐CMC:CPM‐HA) has shown to be safe but needs more data		✓	
	The blending of CaHA‐CMC and CPM‐HA alters the rheologic characteristics compared to the individual filler. Therefore, there is a need for more published evidence regarding the rheologic and biostimulatory properties of the hybrid dermal filler CaHA‐CMC:CPM‐HA	✓		
	There is limited but growing clinical data available regarding the efficacy, safety, and longevity of CaHA‐CMC:CPM‐HA hybrid dermal fillers	✓		

Abbreviations: CaHA‐CMC, calcium hydroxylapatite‐carboxymethyl cellulose; CaHA‐CMC: CPM‐HA, calcium hydroxylapatite‐carboxymethyl cellulose: cohesive polydensified matrix‐hyaluronic acid; CPM‐HA, cohesive polydensified matrix‐hyaluronic acid; SCMM, sternocleidomastoid muscle.

### 3.4 Delphi Panel Guidance on Real‐World Practice Using CaHA‐CMC:CPM‐HA Hybrid Dermal Fillers

Panelists were aligned on nomenclature, treatment indications, efficacy, administration techniques, and regions, the benefits of CaHA‐CMC:CPM‐HA hybrid dermal fillers, safety and contraindications, and existing evidence gaps. The key findings from the study are summarized in Table 3. Details for each category of statements are included below.

**TABLE 3:** Summary of the key findings from the Delphi panel consensus.

Nomenclature	“Hybrid Concentrate” (no additional “fluid”) for 1 syringe CaHA/CMC (1.5 mL) + 1 syringe CPM‐HA (1.0 mL) “Hybrid” (0.5 mL “fluid”) for 1 syringe CaHA/CMC (1.5 mL) + 1 syringe CPM‐HA (1.0 mL) + lidocaine (0.5 mL) “Hybrid Dilute” (1.5 mL “fluid”) for 1 syringe CaHA/CMC (1.5 mL) + 1 syringe CPM‐HA (1.0 mL) + lidocaine (0.5 mL) + Saline (up to 1.5 mL) “Hybrid Hyperdilute” (more than 3.0 mL “fluid”) for 1 syringe CaHA/CMC (1.5 mL) + 1 syringe CPM‐HA (1.0 mL) + lidocaine (0.5 mL) + Saline (at least 2.5 mL)
Main indications + ratios	Skin laxity + Volumization (1:1 CaHA/CMC:CPM‐HA 26 mg/mL → 1:2 for higher volume loss) Contouring (1:1 CaHA/CMC:CPM‐HA 25.5 mg/mL → 2:1 for more laxity) Skin quality (CaHA/CMC:CPM‐HAG 20 mg/mL or CaHA/CMC:CPM‐HA 22.5 mg/mL ratio of 1:3 hyperdilute [i.e., 1.5 mL CaHA, 1.0 mL CPM‐HA, 0.5 mL lidocaine, which is further diluted with saline to reach 1:3 ratio, a total of 6.0 mL] or dilute [i.e., 1.5 mL CaHA, 1.0 mL CPM‐HA, 0.5 mL lidocaine, up to 1.5 mL saline])
Mixing	10 times mix, then 2 times foam, then 10 times mix. One back and forth maneuver is considered to be a single mix. “Foaming” technique increases homogeneity
Layer	Subcutaneous
Benefits	Synergistic effect treating multiple indications with a single injection Value for patients: both immediate and long‐term effects A safe combination, but may not be advised for injection into areas with repetitive muscle activity

Abbreviations: CaHA‐CMC, calcium hydroxylapatite‐carboxymethyl cellulose; CaHA‐CMC: CPM‐HA, calcium hydroxylapatite‐carboxymethyl cellulose: cohesive polydensified matrix‐hyaluronic Acid; CPM‐HA, cohesive polydensified matrix‐hyaluronic acid.

#### 3.4.1 Nomenclature for CaHA‐CMC:CPM‐HA Hybrid Dermal Fillers

The agreed nomenclature statements are shown in the box below. The single statement in the nomenclature category only reached agreement following considerable discussion and debate among the experts, demonstrating the challenges in finding the appropriate terms for each of the different dilutions.

**Table:** 

Nomenclature “Hybrid Concentrate” (no additional “fluid”) for 1 syringe CaHA‐CMC (1.5 mL) + 1 syringe CPM‐HA (1.0 mL) “Hybrid” (0.5 mL “fluid”) for 1 syringe CaHA‐CMC (1.5 mL) + 1 syringe CPM‐HA (1.0 mL) + lidocaine (0.5 mL) “Hybrid Dilute” (1.5 mL “fluid”) for 1 syringe CaHA‐CMC (1.5 mL) + 1 syringe CPM‐HA (1.0 mL) + lidocaine (0.5 mL) + saline (up to 1.0 mL) “Hybrid Hyperdilute” (more than 3.0 mL “fluid”) for 1 syringe CaHA‐CMC (1.5 mL) + syringe CPM‐HA (1.0 mL) + lidocaine (0.5 mL) + saline (at least 2.5 mL)

#### 3.4.2 Treatment Indications and Efficacy of CaHA‐CMC:CPM‐HA Hybrid Dermal Fillers

The panelists agreed that hybrid dermal fillers CaHA‐CMC:CPM‐HA can be used to provide volumization and reshaping of localized areas (e.g., contouring), skin tightening of face, neck, and hands, and to improve skin quality and laxity. This combination can have a synergistic effect both immediately and in the long term by compensating for potential early volume loss due to degradation and reabsorption of the CMC gel. Furthermore, the association of CaHA‐CMC:CPM‐HA can combine neocollagenesis, elastin production, and angiogenesis, while also enhancing volumization and tissue softness. Panelists noted that other factors must also be considered, including that the dilution of CaHA‐CMC:CPM‐HA in saline or lidocaine decreases the G' and viscosity of the original formulation. The dual role of the addition of lidocaine was also highlighted, lidocaine is used both to provide comfort for the patient but can also increase the smoothness and malleability of CaHA‐CMC:CPM‐HA.

#### 3.4.3 Administration Techniques for CaHA‐CMC:CPM‐HA Hybrid Dermal Fillers

**FIGURE 3:**
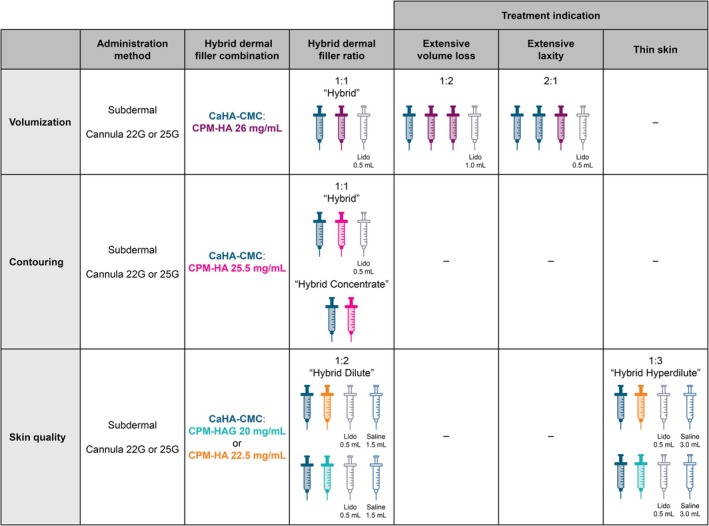
Administration recommendations for CaHA‐CMC:CPM‐HA hybrid dermal fillers. CaHA‐CMC, calcium hydroxylapatite‐carboxymethylcellulose; CPM‐HA, cohesive polydensified matrix‐hyaluronic acid; CPM‐HAG, cohesive polydensified matrix‐hyaluronic acid‐glycerol; Lido, lidocaine.

The statements in this category provided guidance on preparation techniques and the best hybrid mixtures for volumization, contouring, and improving skin quality. Recommendations for achieving a homogeneous hybrid dermal filler include the use of 20 passes when mixing, and the use of the “foaming” technique. This technique involves combining CaHA, HA, and lidocaine, then repeatedly applying negative pressure suction to create a foam‐like consistency, followed by additional mixing passes [13]. Subdermal injection using a 22G or 25G cannula is recommended for all applications. For volumization, CaHA‐CMC:CPM‐HA 26 mg/mL hybrid mixture is recommended, with the optimal ratio dependent on the degree of volume loss and skin laxity. A CaHA‐CMC:CPM‐HA 25.5 mg/mL hybrid mixture is preferred for contouring while hybrid dilute or hyperdilute CaHA‐CMC:CPM‐HA‐(glycerol [G]) 20 mg/mL or CaHA‐CMC:CPM‐HA 22.5 mg/mL are recommended for improvements of skin quality (note: product formulations vary by region, specifically, CPM‐HA 26 mg/mL and CPM‐HA 25.5 mg/mL may or may not contain lidocaine depending on the country or region of distribution; all versions can be used for hybrid formulations, provided appropriate mixing techniques are followed) (Table 2, Figure 3).

**Table:** 

Administration techniques
Preparation To achieve a homogenous hybrid dermal filler CPM‐HA, CaHA‐CMC and lidocaine (ratio dependent on indication) need to be mixed 20 passes The “foaming” technique used to prepare the CaHA‐CMC:CPM‐HA hybrid dermal filler increases product homogeneity and helps with product distribution in the tissue
Volumization For volumization, a 1:1 CaHA‐CMC:CPM‐HA 26 mg/mL hybrid mixture (with 0.5 mL of lidocaine added to the mix) may be administered For volumization hybrid dermal filler CaHA‐CPM‐HA 26 mg/mL is administered via a subdermal injection using a 22G or 25G cannula For cases with minimal or little volume loss and laxity, the CaHA‐CPM‐HA 26 mg/mL mix can be injected with 1:1 ratio for facial lifting to provide subtle enhancement while maintaining product efficiency When treating a more extensive volume loss (with limited laxity), the ratio of the CaHA‐CPM‐HA 26 mg/mL mixture increases up to 1:2 for facial volumization When treating a more extensive laxity, the ratio of the CaHA‐CMC:CPM‐HA mixture can be increased up to 2:1
Contouring For contouring, a 1:1 CaHA‐CMC:CPM‐HA 25.5 mg/mL hybrid mixture (with 0.5 mL of lidocaine added to the mix) may be administered For contouring, hybrid dermal filler CaHA‐CMC:CPM‐HA 25.5 mg/mL is administered via subdermal injection using a 22G or 25G cannula For smaller contour corrections, CaHA‐CMC:CPM‐HA 25.5 mg/mL ratio of 1:1 is applied which is sufficient to achieve the desired aesthetic outcome without overcorrection
Improvement of skin quality The use of CaHA‐CMC:CPM‐HAG 20 mg/mL hybrid dilute or hyperdilute (addition of 1.5–4.5 mL saline) is a suitable filler to improve skin quality For skin quality improvement, hybrid hyperdilute filler CaHA‐CMC:CPM‐HAG 20 mg/mL or CaHA‐CMC:CPM‐HA 22.5 mg/mL is administered via a subdermal injection in multiple directions using either a 22G or 25G cannula For skin quality improvements and areas of thin tissue envelopes (e.g., neck, décolleté), a CaHA‐CMC:CPM‐HAG 20 mg/mL or CaHA‐CMC:CPM‐HA 22.5 mg/mL ratio of 1:3 hyperdilute (i.e., 1.5 mL CaHA, 1.0 mL CPM‐HA, 0.5 mL lidocaine, which is further diluted with saline to reach 1:3 ratio, a total of 6.0 mL) may be applied

#### 3.4.4 Injection Region for CaHA‐CMC:CPM‐HA Hybrid Dermal Fillers

The Delphi panelists agreed on recommendations for subcutaneous injection of hybrid dermal fillers into specific areas for skin quality improvement, volumization and contouring (Table 2).

#### 3.4.5 Patient Benefits of CaHA‐CMC:CPM‐HA Hybrid Dermal Fillers

The panelists agreed that hybrid dermal fillers offer convenience due to their administration in a single injection. There is increased demand for their use among clinicians as a technique to induce biostimulation, reduce facial wrinkles, and improve facial volume and contours. Their use is recommended in men and women seeking a multidimensional effect to multiple tissue structures or layers for volumization, contouring, and skin rejuvenation.

#### 3.4.6 Safety and Contraindications of CaHA‐CMC:CPM‐HA Hybrid Dermal Fillers

The panelists agreed that CaHA‐CMC and CPM‐HA are safe in combination as a hybrid dermal filler. They emphasized that as with CaHA‐CMC alone, injection into areas with repetitive muscle activity, such as the perioral and periorbital regions, is not advised, and that a slow retrograde technique, avoiding bolus injections, should be used to limit post‐injection implant site nodules.

#### 3.4.7 Evidence Gaps Around the Use of CaHA‐CMC:CPM‐HA Hybrid Dermal Fillers

The Delphi panelists noted the lack of standardization in the application of hybrid dermal fillers. This may lead to variations in treatment outcomes and an increased potential for complications. It was also recognized that there is limited but growing clinical data available regarding the efficacy, safety, and longevity of CaHA‐CMC:CPM‐HA hybrid dermal fillers. Although the safety of the individual components of the hybrid dermal filler has been well‐established, the long‐term safety of the hybrid dermal filler itself requires additional data. In addition, the alterations in the rheological characteristics of CaHA‐CMC and CPM‐HA when blended have been recognized, but further published evidence regarding the rheologic and biostimulatory properties of the hybrid dermal filler CaHA‐CMC:CPM‐HA is required. Overall, there is a need for multicenter prospective trials to complement the findings from this Delphi panel.

## 4 Discussion

In this modified Delphi panel study, a notably high level of consensus was achieved early in the process, with panelists agreeing that hybrid dermal fillers add value for patients due to their ability to provide aesthetic improvements, with short recovery times, at a low cost. The panelists noted increased demand among clinicians and patients but acknowledged a lack of standardization in the overall application of hybrid dermal fillers, leading to variations in treatment outcomes and an increased potential for complications. To address these challenges, the panel reached early consensus on best‐practice guidelines on the use of CaHA‐CMC:CPM‐HA hybrid dermal fillers. They also agreed on basic safety, and efficacy requirements for volumization, contouring, and skin quality improvement, as well as identifying gaps in scientific evidence. A high level of agreement between panelists was observed throughout with most (34 out of 43) statements agreed upon in early rounds, illustrating the high level of concordance between panelists. The findings from this Delphi panel are supported by other published papers that have sought to provide guidance regarding filler use [14, 15].

### 4.1 CaHA‐CMC:CPM‐HA Hybrid Dermal Filler Nomenclature, Treatment Administration, and Injection Region

Given the wide range of possible combinations when mixing CaHA‐CMC with the CPM‐HA range, one of the major goals of this study was to establish consensus on hybrid nomenclature, and to identify optimal combinations for each indication. For alignment on nomenclature, the panel defined four categories, based on a 1:1 CaHA‐CMC:CPM‐HA syringe ratio (Figure 3):

- “Hybrid Concentrate” (without lidocaine or saline)
- “Hybrid” (with 0.5 mL lidocaine, without saline)
- “Hybrid Dilute” (with 0.5 mL lidocaine and ≤ 1.5 mL saline)
- “Hybrid Hyperdilute” (with 0.5 mL lidocaine and > 2.5 mL saline)

Some panelists expressed a preference for the formulation containing 0.5 mL of lidocaine, in line with the nomenclature for CaHA‐CMC. This preference was particularly noted for volumization treatments rather than contouring, reporting that this might help reduce the risk of injection technique‐related issues, such as non‐inflammatory nodules and irregularities, a view that was also discussed in the aforementioned advisory board meetings. The use of saline in skin improvement indications has been observed to be more variable, making it challenging to incorporate into a consistent protocol. To establish a more structured approach for these hybrid blends, the panelists agreed to align with earlier hybrid publications that based their dilution strategies on the CaHA dilute and hyperdilute protocols [16]. Following this model, the hybrid blends are generally formulated using unit volumes of 1.5 mL. The standard “Hybrid” blend consists of two 1.5 mL units: 1.5 mL of CaHA combined with 1.0 mL of CPM‐HA and 0.5 mL of lidocaine. The “Hybrid Dilute” blend includes up to an additional 1.5 mL of saline. When more than 1.5 mL of saline is added, the formulation is referred to as “Hybrid Hyperdilute”. The statements in the administration techniques category provide detailed guidance on the use of CaHA‐CMC:CPM‐HA hybrid dermal fillers for a range of aesthetic purposes. Subdermal administration was emphasized as a key factor in achieving optimal outcomes, along with the use of the mixing technique known as “foaming” to enhance product homogeneity [13]. Foaming is a sophisticated procedure, adopted by more than half of the panelists, which represents a significant advance in optimizing hybrid injectable preparation. This method leads to a hybrid dermal filler with significantly improved homogeneous consistency and smoother particle surfaces, which may result in improved aesthetic outcomes and reduced administration problems. The efficacy of this technique has been substantiated by clinical experience and supported by imaging studies. Sonographic analyses of foam‐mixed hybrid injectables demonstrate more defined borders and distinct tissue integration patterns compared to non‐foamed preparations. In addition, electron microscopy reveals smoother particle surfaces and a more uniform injectable structure following foaming. Clinically, these properties translate into more predictable outcomes, particularly in challenging anatomical regions such as the jawline and neck. The incorporation of the foaming technique, combined with a deeper understanding of facial anatomy and injectable rheology, allows practitioners to achieve higher levels of safety, efficacy, and patient satisfaction [13]. The value of the foaming technique was also reinforced in the previously mentioned advisory board meeting (*Advisory board expert opinion, unpublished*).

The panel also discussed the optimal dilution ratios for promoting collagen regeneration. For most volumization or contouring procedures, a 1:1 dilution was considered ideal to benefit from both the volumization/contouring capacity, as well the collagen stimulation; however, for more severe cases or for skin quality improvement, the dilution ratios might vary (Table 3). To reflect the versatility of hybrid dermal fillers, their use was categorized into three primary applications: (1) volumization, (2) contouring, and (3) skin quality improvement. For indications where improvement of skin quality is the focus, the choice of a less concentrated CPM‐HA, such as 20 mg/mL or 22.5 mg/mL, with the addition of saline, significantly lowers the volumizing potential and the G′ of the mixture; therefore, hybrid dilute and hybrid hyperdilute blends will often be the preferred choice for skin quality improvement. In contrast, when volumization or sharp contouring is the goal, a mixture using hybrid blends CPM‐HA 26 mg/mL or 25.5 mg/mL without saline is preferred. Further differentiation was made by the Delphi panel by noting a preference for the blend with CPM‐HA 25.5 mg/mL when sharp contours were required when treating areas such as the jawline.

The final set of statements in the injection region category identified multiple anatomical regions of the face and neck as potential areas suitable for treatment with hybrid dermal fillers. However, the panel agreed that the use of hybrid blends in the hyperdynamic periorbital and perioral areas, as well as the nose, was not recommended. This recommendation is consistent with guidance from Braz et al., who proposed guiding principles and recommendations for the use of CaHA‐HA fillers, which accorded with the findings of this study in recommending subdermal injection, suggesting the avoidance of areas with frequent movement to reduce the risk of nodule formation, and the avoidance of bolus injections [14]. Van Loghem and colleagues similarly recommended the injection of small aliquots of filler when injecting subdermally and provided anatomical recommendations to avoid complications and achieve optimal results when treating different areas [15].

### 4.2 Treatment Indications and the Synergistic Benefits of CaHA‐CMC: CPM‐HA Hybrid Dermal Fillers

Within the treatment indications and efficacy category, agreement was reached on statements regarding the uses of hybrid dermal fillers, their synergistic effects, and rheological properties. The panel affirmed the efficacy of CaHA‐CMC:CPM‐HA in providing volume restoration, contouring, and improved skin quality. These benefits are attributed to a synergistic mechanism that provides both immediate and long‐term benefits, consistent with findings from published studies [8, 9]. The use of HA can provide an immediate and noticeable filling, and lifting effect; however, HA is less effective for increasing dermal thickness and improving skin texture [14, 17]. CaHA, however, has been shown to result in neocollagenesis, and the combination of both HA and CaHA as a hybrid dermal filler has been associated with high levels of patient satisfaction through the restoration of volume, improved skin architecture, and enhanced soft‐tissue quality [14] as well as efficacy in correcting age‐related facial changes with prolonged effects [17]. Histological analyses further support these outcomes, revealing notable dermal remodeling, with CaHA‐CMC:CPM‐HA hybrid dermal fillers, including increased levels of collagen types I and III, an increase in elastic fibers, and elevated epithelial cell proliferation [17].

Understanding the rheological properties of hybrid dermal fillers and how these change when products are mixed is essential for optimizing clinical performance, injection planes, and treatment areas and are the focus of ongoing studies to evaluate the long‐term properties of these products in combination [18]. Hybrid CaHA‐CMC:CPM‐HA dermal fillers exhibit a consistent, volume‐dependent strengthening of their viscoelastic network compared with CPM‐HA alone. This effect is influenced by the HA formulation, mixing ratio, and whether saline is included. Rheological parameters such as G′ increase with CaHA addition, while dilution with saline (e.g., 1:1 to 1:4) reduces stiffness but does not eliminate the reinforcing effect of CaHA. Adding saline or lidocaine further softens the blend, making it more fluid and less cohesive. However, these hybrids behave differently from simple CaHA dilutions due to the contribution of the HA matrix. These rheological changes affect injectability and tissue integration: stiffer, more cohesive blends are suited for deep volumization and contouring, while softer, more spreadable formulations are ideal for biostimulation and superficial skin quality enhancement [18].

### 4.3 Safety and Remaining Evidence Gaps in the Use of CaHA‐CMC: CPM‐HA Hybrid Dermal Fillers

The advantages of CaHA‐CMC: CPM‐HA hybrid dermal fillers compared with commercially available hybrid products have been increasingly recognized, with CaHA‐CMC:CPM‐HA hybrid dermal fillers demonstrated to offer a customizable approach to treatment according to patient needs through choice of HA filler with different rheological properties and dilutions [10]. However, safety and efficacy depend on the products chosen. The panelists agreed that CaHA‐CMC and CPM‐HA are a safe combination and highlighted that both products have individually well‐established safety and efficacy profiles. CaHA‐CMC has been reported in previous studies to have a good safety profile; with no long‐term or delayed‐onset adverse events reported [19, 20]. Although CaHA fillers do not have a direct enzymatic antidote, unlike HA fillers which can be dissolved with hyaluronidase, CaHA has long been used in non‐hybrid forms with a well‐established safety profile, and the absence of a reversal agent has not hindered its widespread clinical adoption [21]. Likewise, safety has been demonstrated with CPM‐HA use in multiple studies, with a similar profile of a small number of reported mild adverse events, resolved without intervention [22, 23, 24, 25, 26]. Furthermore, the combination of CaHA‐CMC and CPM‐HA is, to date, the only filler mix which has published safety and efficacy data, again revealing a good safety profile [27].

A 5‐year retrospective analysis of treatments with CaHA‐CMC:CPM‐HA hybrid dermal filler in 2112 patients reported only five minor adverse events (0.24%). These comprised four non‐inflammatory nodules (0.19%), of which two completely resolved following the use of hyaluronidase, and one case of transient edema [28]. These findings demonstrate the favorable safety profile of the CaHA‐CMC:CPM‐HA hybrid dermal filler and align with vascular safety data from dilutional rheology studies of CaHA‐CMC which demonstrated that a titrated dilution could be used to tailor the rheological properties of the product and thus expand the range of available clinical applications while decreasing the risk of vascular events [10, 29]. Furthermore, a recent clinical study revealed that diluted (1:1) and hyperdiluted (1:2) CaHA‐CMC can achieve improved overall efficacy in remodeling the extracellular matrix with a lower immune response, compared with poly‐l‐lactic acid [30]. The panel concluded that although the body of evidence supporting the safe and effective use of hybrid dermal fillers is growing, there is a need for more data, including evidence supporting the rheologic and biostimulatory properties of the CaHA‐CMC:CPM‐HA hybrid dermal filler.

### 4.4 Study Strengths and Limitations

The current study demonstrated several strengths, primarily the use of the modified Delphi panel design, which is recognized as a validated procedure for achieving consensus. This systematic and structured process is widely used to harness expert opinion and address fundamental questions in healthcare. The early consensus reached across the majority of statements illustrates the high level of agreement among experts in this field on the use of hybrid dermal fillers and reinforces the validity of the panel's recommendations on standardizing nomenclature and mixing techniques. Notwithstanding, there are some limitations that should be considered when interpreting the results. The panel included participants from various geographical regions, and so cross‐country differences may hinder the achievement of consensus. These geographical locations were also limited to regions where the component for mixing dermal fillers were available for use, which may have reduced the generalizability of the results to all populations. Despite this, no regional disparities were observed, underscoring the importance of the Delphi panel consensus methodology and the universal applicability of published guidelines. In addition, there are limited published data on the use of CaHA‐CMC:CPM‐HA hybrid dermal fillers, with most of the existing body of literature being published in parallel with conduct of the Delphi process, highlighting a paucity of literature for the panel to review and debate and illustrating the importance of studies such as this Delphi panel in providing guidance on standardized use of these products. Additionally, this was an unblinded study, therefore, all HCPs involved were aware of the role of the study sponsor and had been selected from a sponsor‐provided list to ensure relevant expertise. While this approach ensured informed contributions, it may have introduced bias into participant responses.

Finally, although the findings are considered robust, as the discussions permitted by the experts available for Round 3 outweighed the relatively small sample size, cross‐country comparisons were not possible. Although the ideal number of participants for a Delphi panel remains an ongoing issue of debate, panels as small as four participants have been considered appropriate, and the ideal range of participants has been suggested to be between eight and 23 [11]. The high degree of early consensus observed on the majority of statements and the six participants involved in Round 3 of the current study were considered sufficient to permit a robust consensus to be reached, particularly given the discursive nature of this round of the study.

## 5 Conclusion

The findings from this study provide a strong foundation for standardized practices in the application of CaHA‐CMC:CPM‐HA hybrid dermal fillers. Agreement was reached by the Delphi panel on key aspects of the use of hybrid dermal fillers, including indications, efficacy, administration techniques, injection regions, and safety. This guidance agreed on by the panel can support practitioners in delivering effective aesthetic treatments and can play a critical role in enhancing patient safety by minimizing variability in clinical practice. Use of this expert‐led and evidence‐based guidance by aesthetic HCPs will ultimately help ensure that patients receive high‐quality, safe, and predictable aesthetic treatments, regardless of provider or setting.

## Author Contributions

All authors were involved in the conception and/or design of the work, acquisition of data, data analysis, and/or interpretation of data. All authors had access to the study results, reviewed and revised the manuscript, and approved the final draft submitted for publication.

## Funding

This study was sponsored by Merz Aesthetics GmbH.

## Ethics Statement

The authors have nothing to report.

## Conflicts of Interest

J.K. has received consulting fees, payment or honoraria for lectures, presentations, speakers bureaus, manuscript writing or educational events, and support for attending meetings, as well as participating in data safety monitoring or advisory board activities for Merz Aesthetics (Frankfurt, Germany). R.B. declares no conflicts of interest. N.F.‐G. is a consultant for Merz Aesthetics (Frankfurt, Germany). O.H. declares no conflicts of interest. M.K. received financial support for clinical trials with University of Hamburg, Cosmetic Science Division from Allergan Abbvie, Croma, and Ipsen Innovation, as well as consulting fees, payment or honoraria for lectures, presentations, speaker bureaus, manuscript writing or educational events, and participation in data safety monitoring or advisory board activities for Merz Aesthetics (Frankfurt, Germany), Allergan Abbvie, Ipsen Innovation, Croma, Nordberg Medical, and Neauvia. L.P. has received consulting fees, and payment for expert testimony from Merz Aesthetics (Frankfurt, Germany) and Vydence Laser. In addition, L.P. has received payment or honoraria for lectures, presentations, manuscript writing or educational events, and support for attending meetings, as well as participating in data safety monitoring or advisory board activities for Merz Aesthetics (Frankfurt, Germany). R.V. has received speaker fees from Merz Aesthetics (Frankfurt, Germany). Y.Y. declares no conflicts of interest. C.d.S.P. has received consulting fees, payment or honoraria for lectures, presentations, speakers bureaus, manuscript writing or educational events, and support for attending meetings, as well as participating in data safety monitoring or advisory board activities for Merz Aesthetics (Frankfurt, Germany).

## Data Availability

All data relevant to the study are included in the article.

## References

[1] L. Triana , R. M. Palacios Huatuco , G. Campilgio , and E. Liscano , “Trends in Surgical and Nonsurgical Aesthetic Procedures: A 14‐Year Analysis of the International Society of Aesthetic Plastic Surgery‐ISAPS,” Aesthetic Plastic Surgery 48, no. 20 (2024): 4217–4227. 39103642 10.1007/s00266-024-04260-2

[2] International Society of Aesthetic Plastic Surgeons , Global Survey 2023 (International Society of Aesthetic Plastic Surgeons (ISAPS), 2023).

[3] B. J. Mahmood Faris , “The Use of Facial Fillers in Clinical Practice: The Level of Patient Satisfaction and an Overview of Common Clinical Complications,” Actas Dermo‐Sifiliográficas 115, no. 5 (2024): 458–465. 37865230 10.1016/j.ad.2023.10.008

[4] I. Sánchez‐Carpintero , D. Candelas , and R. Ruiz‐Rodríguez , “Dermal Fillers: Types, Indications, and Complications,” Actas Dermo‐Sifiliográficas 101, no. 5 (2010): 381–393. 20525480 10.1016/s1578-2190(10)70660-0

[5] S. B. Aguilera , A. McCarthy , S. Khalifian , Z. P. Lorenc , K. Goldie , and W. G. Chernoff , “The Role of Calcium Hydroxylapatite (Radiesse) as a Regenerative Aesthetic Treatment: A Narrative Review,” Aesthetic Surgery Journal 43, no. 10 (2023): 1063–1090. 37635437 10.1093/asj/sjad173 PMC11025388

[6] N. Fakih‐Gomez , C. Muñoz‐Gonzalez , C. A. Porcar Plana , A. Verano Garcia , and J. Kadouch , “Standarized Protocols for Hybrid Filler Application in Aesthetic Medicine,” American Journal of Cosmetic Surgery 42, no. 3 (2025): 174–180.

[7] A. Mills and S. Haq , “HArmonyCa: A First‐In‐Class, Hybrid, Dual‐Functioning Hyaluronic Acid/Calcium Hydroxyapatite Dermal Filler,” Journal of Aesthetic Nursing 12, no. Suppl 8 (2023): S6–S12.

[8] N. Fakih‐Gomez and J. Kadouch , “Combining Calcium Hydroxylapatite and Hyaluronic Acid Fillers for Aesthetic Indications: Efficacy of an Innovative Hybrid Filler,” Aesthetic Plastic Surgery 46, no. 1 (2022): 373–381. 34341855 10.1007/s00266-021-02479-x PMC8831259

[9] N. Fakih‐Gomez , A. Verano‐Garcia , C. A. Porcar Plana , C. Muñoz‐Gonzalez , and J. Kadouch , “Jawline Sharp Contouring With Hybrid Filler,” Aesthetic Plastic Surgery 49, no. 1 (2025): 334–340. 39014233 10.1007/s00266-024-04226-4

[10] A. D. McCarthy , D. J. Soares , A. Chandawarkar , R. El‐Banna , G. E. de Lima Faria , and N. Hagedorn , “Comparative Rheology of Hyaluronic Acid Fillers, Poly‐l‐Lactic Acid, and Varying Dilutions of Calcium Hydroxylapatite,” Plastic and Reconstructive Surgery‐Global Open 12, no. 8 (2024): e6068. 39148505 10.1097/GOX.0000000000006068 PMC11326459

[11] Z. Shang , “Use of Delphi in Health Sciences Research: A Narrative Review,” Medicine 102, no. 7 (2023): e32829. 36800594 10.1097/MD.0000000000032829 PMC9936053

[12] C.‐C. Hsu and B. A. Sandford , “The Delphi Technique: Making Sense of Consensus,” Practical Assessment, Research & Evaluation 12, no. 10 (2007): 1–8.

[13] N. Fakih‐Gomez , J. Kadouch , Í. Aragón‐Niño , et al., “The Foaming Technique: Innovation in Mixing Calcium Hydroxylapatite Applied in Hybrid Filler,” Aesthetic Plastic Surgery 49 (2025): 5824–5835. 40588639 10.1007/s00266-025-05055-9

[14] A. Braz , C. C. de Paula Eduardo , A. Pierce , A. Grond , A. Kutikov , and L. Nakab , “A Novel Hybrid Injectable for Soft‐Tissue Augmentation: Analysis of Data and Practical Experience,” Plastic and Reconstructive Surgery. Global Open 12, no. 9 (2024): e6190. 39301305 10.1097/GOX.0000000000006190 PMC11412701

[15] J. van Loghem , S. Sattler , G. Casabona , et al., “Consensus on the Use of Hyaluronic Acid Fillers From the Cohesive Polydensified Matrix Range: Best Practice in Specific Facial Indications,” Clinical, Cosmetic and Investigational Dermatology 14 (2021): 1175–1199. 34526796 10.2147/CCID.S311017 PMC8435881

[16] K. Goldie , W. Peeters , M. Alghoul , et al., “Global Consensus Guidelines for the Injection of Diluted and Hyperdiluted Calcium Hydroxylapatite for Skin Tightening,” Dermatologic Surgery 44, no. Suppl 1 (2018): S32–S41. 30358631 10.1097/DSS.0000000000001685

[17] Y. A. Yutskovskaya , “Clinical and Histopathological Evaluation of Skin Changes Following Intradermal Injection of Hybrid Products Combining Calcium Hydroxyapatite With Various Density Hyaluronic Acid,” Madridge Journal of Dermatology & Research 7, no. 1 (2024): 126–135.

[18] E. Kaczuba , N. Fakih‐Gomez , J. Kadouch , et al., “Exploring the Rheology and Clinical Potential of Calcium Hydroxylapatite‐Hyaluronic Acid Hybrids,” Journal of Cosmetic Dermatology 24, no. 10 (2025): e70473. 41081304 10.1111/jocd.70473 PMC12516938

[19] T. Pavicic , “Calcium Hydroxylapatite Filler: An Overview of Safety and Tolerability,” Journal of Drugs in Dermatology 12, no. 9 (2013): 996–1002. 24002146

[20] J. A. Kadouch , “Calcium Hydroxylapatite: A Review on Safety and Complications,” Journal of Cosmetic Dermatology 16, no. 2 (2017): 152–161. 28247924 10.1111/jocd.12326

[21] R. Zago Sá Fortes , J. Cassol Spanemberg , K. Cherubini , and F. G. Salum , “Adverse Events and Satisfaction Outcomes With Calcium Hydroxylapatite and Polycaprolactone Fillers in Facial Aesthetics: A Systematic Review,” Cosmetics 11, no. 5 (2024): 165.

[22] W. Prager , K. Agsten , M. Kravtsov , and P. M. Kerscher , “Mid‐Face Volumization With Hyaluronic Acid: Injection Technique and Safety Aspects From a Controlled, Randomized, Double‐Blind Clinical Study,” Journal of Drugs in Dermatology 16, no. 4 (2017): 351–357. 28403269

[23] H. Buntrock , T. Reuther , W. Prager , and M. Kerscher , “Efficacy, Safety, and Patient Satisfaction of a Monophasic Cohesive Polydensified Matrix Versus a Biphasic Nonanimal Stabilized Hyaluronic Acid Filler After Single Injection in Nasolabial Folds,” Dermatologic Surgery 39, no. 7 (2013): 1097–1105. 23506356 10.1111/dsu.12177

[24] T. Pavicic , G. Sattler , W. Prager , et al., “Safety of Cohesive Polydensified Matrix Cross‐Linked Hyaluronic Acid Volumizing Gel in Temporal Hollows and Cheeks: A Prospective, Open‐Label, Postmarket Study,” Dermatologic Surgery 47, no. 10 (2021): 1359–1364. 34417392 10.1097/DSS.0000000000003176

[25] B. S. Biesman , J. R. Montes , R. C. Radusky , S. Mersmann , and V. W. Graul , “A Prospective, Multicenter, Evaluator‐Blind, Randomized, Controlled Study of Belotero Balance (+), a Hyaluronic Acid Filler With Lidocaine, for Correction of Infraorbital Hollowing in Adults,” Aesthetic Surgery Journal 44, no. 9 (2024): 976–986. 38377391 10.1093/asj/sjae039

[26] D. Hertz‐Kleptow , A. Hanschmann , M. Hofmann , T. Reuther , and M. Kerscher , “Facial Skin Revitalization With CPM((R))‐HA20G: An Effective and Safe Early Intervention Treatment,” Clinical, Cosmetic and Investigational Dermatology 12 (2019): 563–572. 31496779 10.2147/CCID.S209256 PMC6698156

[27] J. Kadouch and N. Fakih‐Gomez , “A Hybrid Filler: Combining Calcium Hydroxylapatite and Hyaluronic Acid Fillers for Aesthetic Indications,” American Journal of Cosmetic Surgery 39, no. 3 (2021): 182–189. 10.1007/s00266-021-02479-x PMC8831259 34341855

[28] N. Fakih‐Gomez , J. Kadouch , F. Felice , D. Haykal , and C. Muñoz‐Gonzalez , “The Hybrid Filler Technique: A 5‐Year Retrospective Analysis,” Aesthetic Plastic Surgery 49, no. 3 (2025): 618–626. 39327280 10.1007/s00266-024-04387-2

[29] A. D. McCarthy , D. J. Soares , A. Chandawarkar , R. El‐Banna , and N. Hagedorn , “Dilutional Rheology of Radiesse: Implications for Regeneration and Vascular Safety,” Journal of Cosmetic Dermatology 23, no. 6 (2024): 1973–1984. 38357772 10.1111/jocd.16216

[30] G. Casabona , “Extracellular Matrix Response to Diluted Calcium Hydroxylapatite Microspheres and Poly‐l‐Latic Acid Microparticles,” International Master Course on Aging Science (2025).

